# Sex- and age-specific normative values for handgrip strength and components of the Senior Fitness Test in community-dwelling older adults aged 65–75 years in Germany: results from the OUTDOOR ACTIVE study

**DOI:** 10.1186/s12877-021-02188-9

**Published:** 2021-04-26

**Authors:** Birte Marie Albrecht, Imke Stalling, Karin Bammann

**Affiliations:** Institute for Public Health and Nursing Sciences (IPP), University of Bremen, Grazer Straße 2a, 28359 Bremen, Germany

**Keywords:** Elderly, Functional fitness, GAMLSS, Germany, Handgrip strength, Normative values, Older adults, Physical fitness, Reference values, Senior fitness test

## Abstract

**Background:**

Physical fitness is a key component of independent living and healthy ageing. For the measurement of physical fitness in older adults, the Senior Fitness Test is a commonly used tool. The objective of this study is to calculate sex- and age-specific normative values for handgrip strength and components of the Senior Fitness Test for older adults (65–75 years) in Germany.

**Methods:**

Cross-sectional data of 1657 community-dwelling older adults residing in Bremen, Germany (53% female) were included in this study. Physical fitness was assessed using the following measurements of the Senior Fitness Test battery: 30s-chair stand test, 2 min-step test, sit-and-reach test, and back scratch test. In addition, handgrip strength was measured using a Saehan DHD-3 digital hand dynamometer SH1003. Sex- and age specific normative values were calculated for the 1st, 3rd, 10th, 25th, 50th, 75th, 90th, 97th, and 99th percentile using the GAMLSS method.

**Results:**

The normative values show differences dependent on sex and age. For handgrip strength, the 30s-chair stand test and the 2 min-step test, normative values were higher for men, while women reached higher values in the sit-and-reach test and the back scratch test. For both, men and women, normative values declined with age.

**Conclusions:**

This study provides sex- and age-specific normative values for handgrip strength and components of the Senior Fitness Test for older adults in Germany. They might be useful for future research and for the application in practice.

**Supplementary Information:**

The online version contains supplementary material available at 10.1186/s12877-021-02188-9.

## Background

Maintaining a high level of physical fitness is required for independent living in the process of ageing [1, 2]. Muscle strength, aerobic endurance, flexibility, and balance are needed for many activities of daily living – for example running errands, carrying groceries, getting dressed, or cleaning. In addition, physical fitness is positively associated with health-related quality of life [3, 4] and well-being [5]. Yet, physical fitness declines with increasing age [6, 7].

The assessment of physical fitness is important in research as well as in practice. One of the standard tools for adults aged 60 years and above is the Senior Fitness Test which was introduced by Rikli & Jones in 1999 [8]. The original version of the Senior Fitness Test includes the 30s-chair stand and arm curl test for measuring lower and upper body strength, the sit-and-reach and back scratch test for measuring lower and upper body flexibility, the 6 min-walk or the 2 min-step test for measuring aerobic endurance, and the 8 ft. up-and-go test for measuring dynamic balance [8]. The test results can be interpreted using normative values, e.g. the ones provided by Rikli & Jones for older adults in the USA in 1999 [9]. Since then, other authors published further normative values for older adults in different parts of the world – for example Taiwan [10], Spain [11, 12], Portugal [13], Hong Kong [14], Chile [15], and Poland [16]. To the best of our knowledge, there are no normative values for the Senior Fitness Test for older adults in Germany.

The objective of this study is to provide sex- and age-specific normative values for handgrip strength and components of the Senior Fitness Test in community-dwelling adults aged 65–75 years in Germany.

## Methods

### Study design and population

The OUTDOOR ACTIVE study is part of the prevention research network AEQUIPA that investigates the role of physical activity as a key determinant of healthy ageing [17]. In this context, the aim of OUTDOOR ACTIVE is to develop and implement a community-based outdoor physical activity promotion program in older adults using a participatory approach. The OUTDOOR ACTIVE study consists of two parts: the pilot study (02/15–01/18) and the cluster-randomized trial (02/18–01/21). The eligibility criteria for both parts were 1) being between 65 and 75 years of age, 2) being non-institutionalized, and 3) living in specific subdistricts in Bremen, Germany (pilot study: Arbergen, Hastedt, Hemelingen, Mahndorf, Sebaldsbrück; cluster-randomized trial: Blumenthal, Burg-Grambke, Gete, Lehe, Lehesterdeich, Neustadt, Ohlenhof, Ostertor). Address data were obtained from the registry office of Bremen and eligible individuals were initially contacted via letter.

The data for the calculation of normative values were obtained during the baseline assessments of the pilot study and the cluster-randomized trial [18]. The participation in the baseline assessment included 1) a questionnaire on intrapersonal, interpersonal, and environmental determinants of physical activity, 2) a health examination including a short physical examination and a fitness test, and 3) a seven-day accelerometer measurement. The follow-up assessment took place 1 year after baseline, yet the data were not included in the following analyses.

In total, 11,079 individuals meeting the age criteria were registered in the study regions of the pilot study and the cluster-randomized trial. Of those, 461 could not participate due to acute health problems and 125 deceased. Four hundred fifty individuals moved outside the study region and 77 could not participate because of language barriers. Of the remaining 9966 confirmed eligible individuals, 3425 were never reached and 4247 refused to participate. Furthermore, 151 individuals of the subdistrict Lehesterdeich were never contacted because the end of the Lehesterdeich survey period was reached and the actual sample size of the subdistrict already exceeded the calculated sample size. Two thousand one hundred forty-three individuals participated in at least one part of the pilot study or the cluster-randomized trial and, of those, 1657 participants completed at least one physical fitness measurement and were, therefore, included in the calculation of normative values.

All participants provided written informed consent. Both the pilot study and the cluster-randomized trial were approved by the ethical committee of the University of Bremen.

### Measures

The health examinations to collect data on physical fitness, anthropometry, and age took place in survey rooms in the respective subdistricts between 10/15–08/16 (pilot study) and 06/18–07/19 (cluster-randomized trial). The examinations were conducted by trained survey personnel.

The implementation of the Senior Fitness Test by Rikli & Jones [8] to assess the physical fitness of the OUTDOOR ACTIVE participants is depicted in Table 1. Handgrip strength – measured with a Saehan DHD-3 digital hand dynamometer SH1003 (Saehan Corporation, Changwon, South Korea) – was used in lieu of the 30s-arm curl test to assess upper body strength. The measurement was conducted in a standing position, upper arm close to the upper body, and elbow flexed in a 90° angle. Maximum isometric strength was measured twice for both hands and the overall maximum was used for the calculation of normative values [21]. For the 30s-chair stand test, the participant has to stand up from a seated position and sit back down as often as possible in 30s. The 2 min-step test requires the participant to step in place for 2 min with both knees reaching a required height. During the sit-and-reach test, the participant sits on a chair with one leg extended and has to reach toward his toes. For the back scratch test, the participant has to try to touch both hands behind the back with one hand reaching over the shoulder and the other up the middle of the back. The tests were conducted according to the test protocols in the Senior Fitness Test Manual, 2nd edition [19]*.* As depicted in Table 1, the 4-stage balance test [20] to assess static balance was used in lieu of the 8 ft. up-and-go test as a measure of dynamic balance. For the 4-stage balance test, the participant has to hold four different positions with increasing level of difficulty for 10s. As the result variable is binary (yes/ no for each position), we could not calculate any normative values.

**Table 1:** Implementation of the Senior Fitness Test [19] in the OUTDOOR ACTIVE study

Physical fitness domain	Senior Fitness Test	OUTDOOR ACTIVE
Upper body strength	30s-arm curl test	Handgrip strength
Lower body strength	30s-chair stand test	30s-chair stand test
Aerobic endurance	6 min-walk test OR 2 min-step test	2 min-step test
Lower body flexibility	Sit-and-reach test	Sit-and-reach test
Upper body flexibility	Back scratch test	Back scratch test
Dynamic balance	8 ft. up-and-go test	/^a^

^a^Instead of dynamic balance, static balance was assessed using the 4-stage balance test [20]

Height was measured with a Seca 217 mobile stadiometer (Seca GmbH & Co. KG, Hamburg, Germany), body weight with a Kern MPC 250K100M personal floor scale (Kern & Sohn GmbH, Ballingen, Germany), and waist circumference with a Seca 201 measuring tape (Seca GmbH & Co. KG, Hamburg, Germany). Body mass index was calculated as the quotient of body weight (in kg) and squared height (in m) and, afterwards, classified into underweight, normal weight, overweight, and obesity according to the World Health Organization [22].

Sociodemographic information (sex, educational status) and self-rated health were assessed through a self-administered questionnaire. Educational status was categorized according to the International Standard Classification of Education 1997 [23]. Self-rated health was assessed with a single item from the SF-36 questionnaire [24].

### Statistical analyses

For the description of the study population, sex-stratified absolute and relative frequencies of the educational status, self-rated health, and body mass index were determined. Means and standard deviations were calculated for age, body weight, body height, waist circumference, and the physical fitness measurements. Descriptive analyses were performed with SPSS® Statistics version 20.0 (IBM Corp., Armonk, NY, USA).

Generalized additive models for location, scale, and shape (GAMLSS) were used to estimate sex-stratified percentile curves dependent on age for all physical fitness measurements. GAMLSS are an extension of the LMS method [25] and allow not only to model for the mean, variability, and skewness of the response variable but also for kurtosis [26]. For each model, the distribution of the response variable was selected based on Akaike’s information criterion (see Additional file 1). Cubic splines were used for smoothing. Sex- and age-specific percentile curves were plotted for percentiles 1, 3, 10, 25, 50, 75, 90, 97, and 99. The GAMLSS models were calculated and the results plotted using the gamlss package version 5.1–7 [27] in RStudio version 3.6.2 (RStudio, Inc., Boston, MA, USA).

## Results

In total, 880 women (53.1%) and 777 men (46.9%) were included in the calculation of normative values. The mean age of women was 69.6 ± 2.9 years and of men 69.5 ± 2.8 years (for number of participants by sex and age see Additional file 2). The majority of women had normal weight (41.6%) in comparison to men, where the majority was overweight (50.7%). Most women (58.7%) and men (56.4%) declared their health status as good. Educational status was higher in men (advanced education: 64.1%) compared to women (advanced education: 37.3%) (see Table 2). Means, standard deviations, and standard errors by sex and age for all physical fitness measurements are depicted in Additional file 3.

**Table 2:** Characteristics of the study population

Participants’ characteristics	Women (*n* = 880)	Men (*n* = 777)
	n (%)	n (%)
Education		
Basic education (ISCED level 1 + 2)	151 (17.8)	30 (4.1)
Specialized education (ISCED level 3 + 4)	381 (44.9)	235 (31.8)
Advanced education (ISCED level ≥ 5)	316 (37.3)	474 (64.1)
Body-mass-index (kg/m^2^)		
Underweight (< 18.5)	9 (1.0)	0
Normal weight (18.5 - < 25)	364 (41.6)	211 (27.2)
Overweight (25 - < 30)	321 (36.7)	394 (50.7)
Obesity (≥ 30)	181 (20.7)	172 (22.1)
Self-rated health		
Bad	14 (1.7)	10 (1.4)
Less good	126 (15.0)	86 (11.6)
Good	492 (58.7)	417 (56.4)
Very good	175 (20.9)	197 (26.6)
Excellent	31 (3.7)	30 (4.1)
	Mean (SD)	Mean (SD)
Age (years)	69.6 (2.9)	69.5 (2.8)
Body weight (kg)	70.4 (12.8)	85.8 (13.5)
Body height (cm)	162.8 (6.6)	176.5 (6.8)
Waist circumference (cm)	88.2 (12.3)	100.1 (11.8)
Handgrip strength (kg)	25.2 (5.1)	42.0 (7.8)
30s-chair stand test (n in 30s)	12.9 (3.0)	13.4 (3.0)
2 min-step test (n in 2 min)	84.7 (17.7)	87.1 (17.7)
Sit-and-reach test (cm)	3.6 (9.9)	−3.9 (11.3)
Back scratch test (cm)	−4.5 (9.0)	−11.6 (12.4)

*ISCED* International Standard Classification of Education

*SD* standard deviation

**Fig. 1:**
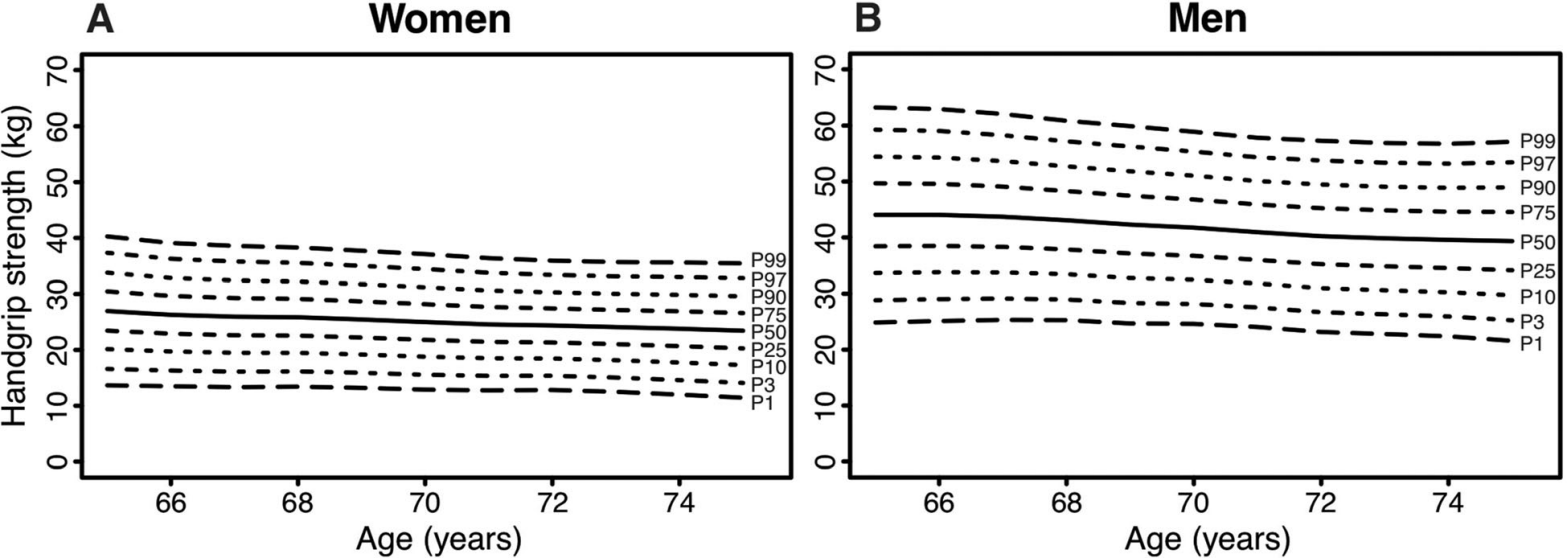
Age-specific normative values for handgrip strength for women (**a**) and men (**b**)

**Fig. 2:**
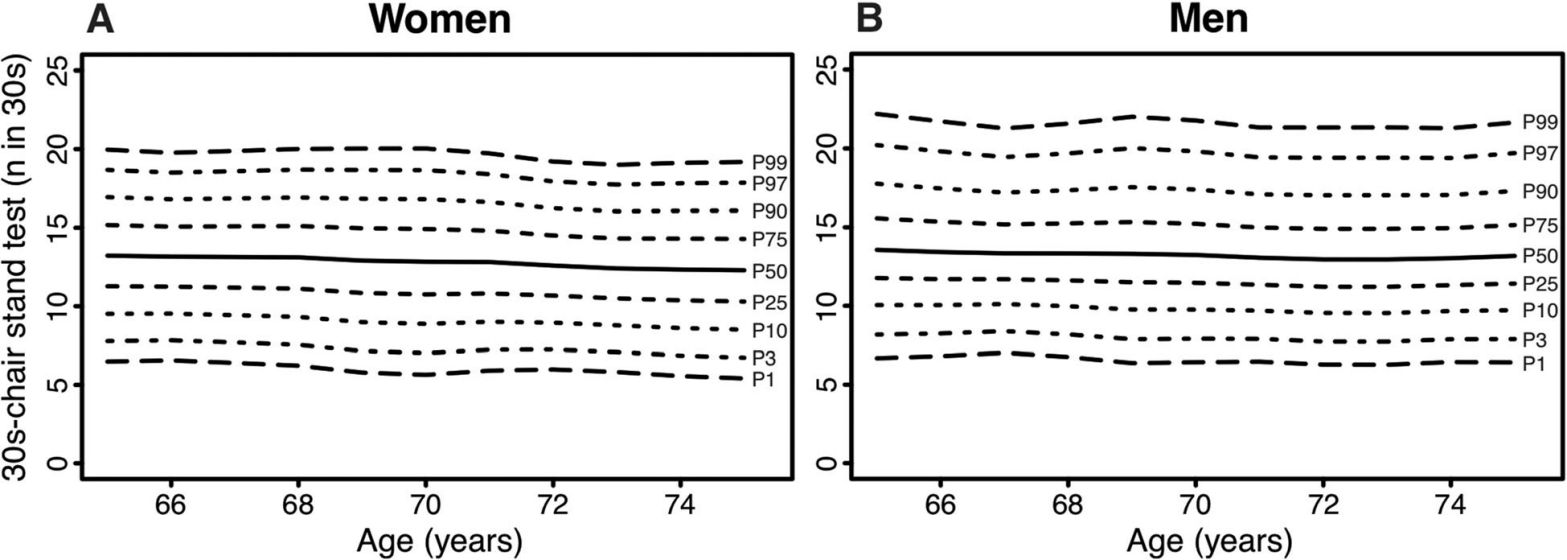
Age-specific normative values for the 30s-chair stand test for women (**a**) and men (**b**)

**Fig. 3:**
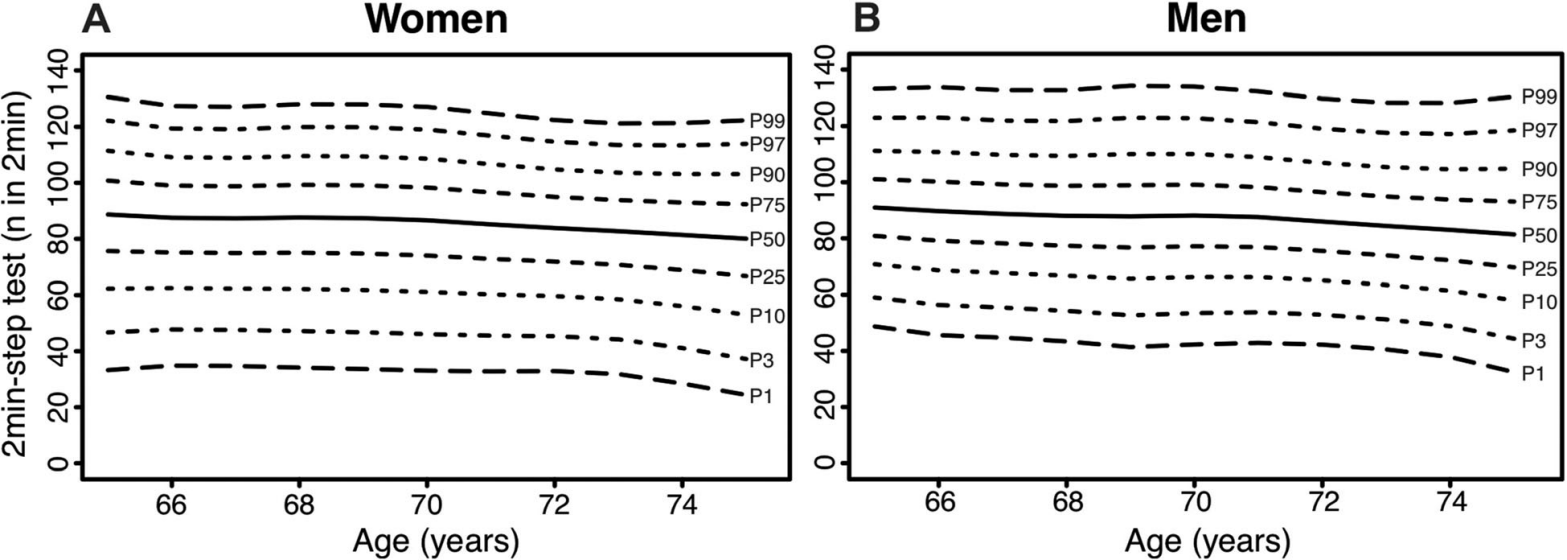
Age-specific normative values for the 2 min-step test for women (**a**) and men (**b**)

**Fig. 4:**
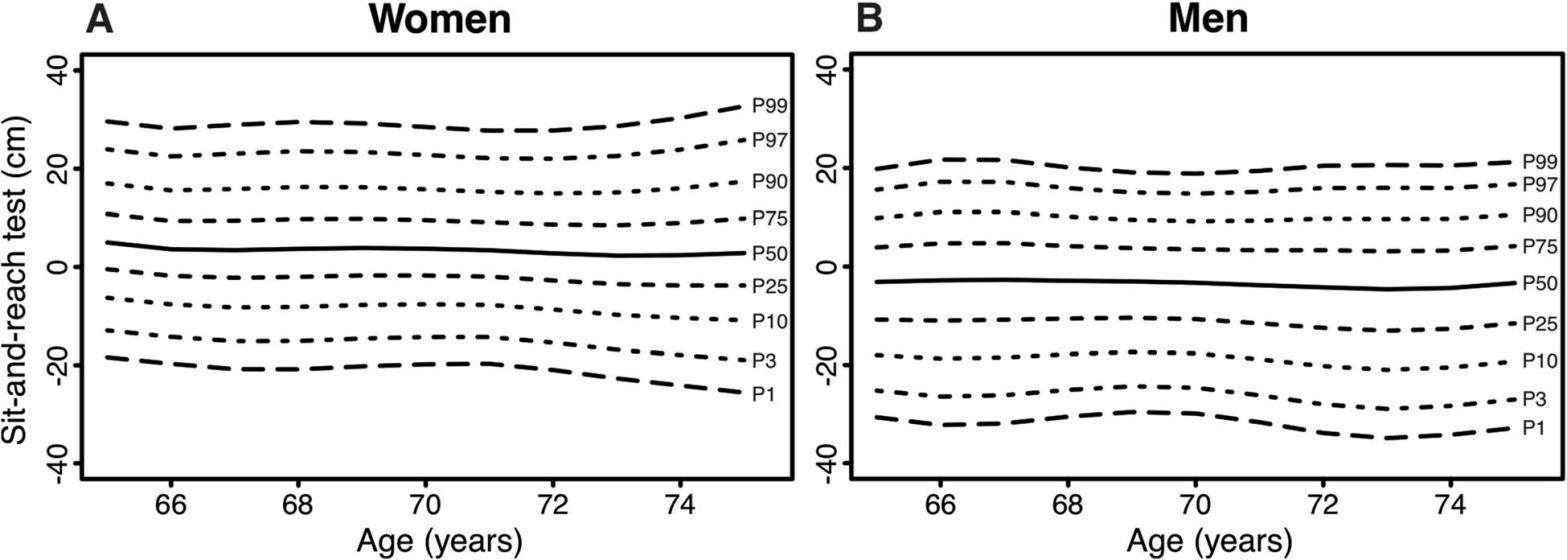
Age-specific normative values for the sit-and-reach test for women (**a**) and men (**b**)

**Fig. 5:**
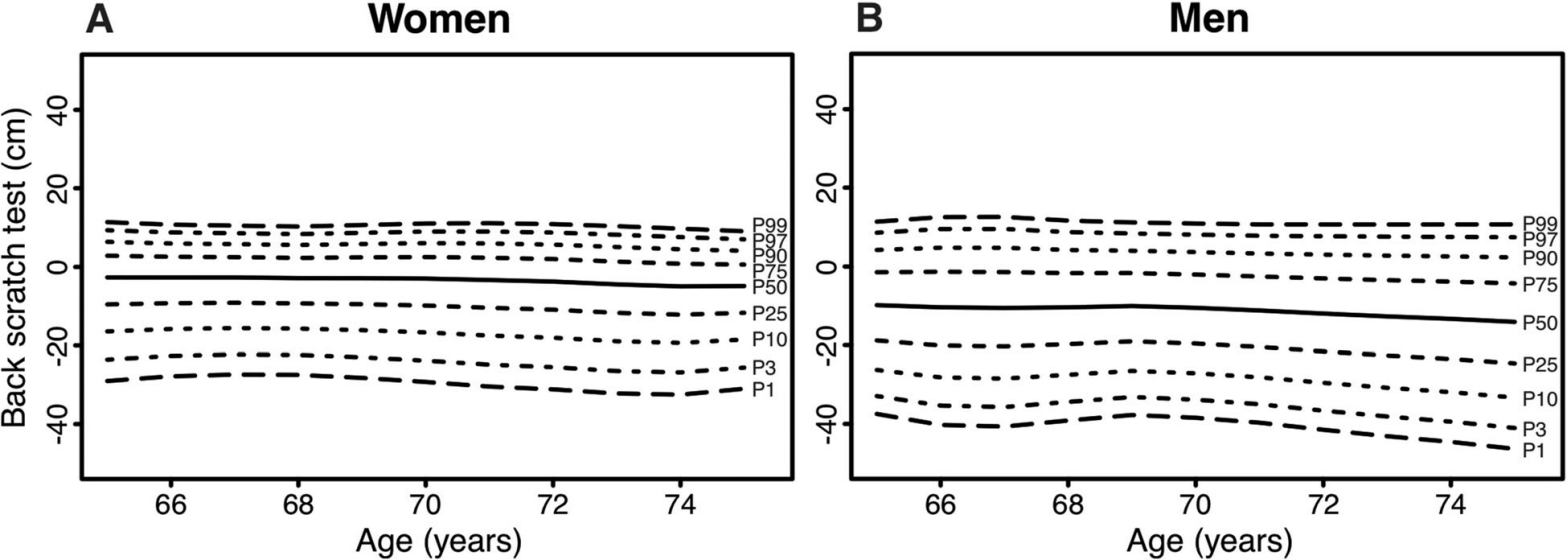
Age-specific normative values for the back scratch test for women (**a**) and men (**b**)

Age- and sex-specific normative values for the physical fitness measurements are displayed in Figs. 1, 2, 3, 4, 5 (for Tables see Additional file 4). Test results differed between women and men across all physical fitness measurements with women reaching higher values in the flexibility domains (sit-and-reach test and back scratch test) and men reaching higher values in the strength and endurance domains (handgrip strength, 30s-chair stand test, and 2 min-step test). Apart from minor exceptions, test results declined with increasing age for both women and men.

## Discussion

This paper provides sex- and age-specific normative values for handgrip strength and components of the Senior Fitness Test. The test results of all physical fitness measurements differed between woman and men, yet the age-specific decline was similar for both.

The sex differences with men performing higher in endurance and muscle strength domains as well as the superiority of women in the flexibility domains are in line with other studies [9–14, 16, 28–31]. This also applies to the age-specific decline in test results [9–16, 28–31]. Yet, specific values differ between studies. The normative values for handgrip strength provided in this paper are overall higher than other published values [14, 28–31]. Handgrip strength is positively correlated to body height [28, 32, 33]. This could be one reason for the differences as the participants in our study were considerably taller. In the 30s-chair stand test and the 2 min-step test, our normative values for both women and men are lower than most of the other values reported [9–11, 13–16]. One possible explanation could be the differences in body weight. Only some studies reported the body weight of their participants, but in most studies that did, the participants weighted less than ours [9, 11, 14, 15]. For the 2 min-step test, only Chung et al. reported lower values [14]. Chen et al. provided similar normative values for the 30s-chair stand test [10]. There is indication that flexibility is dependent on culture [34]. For example, older adults in Hong Kong like to engage in “light Chinese-style mind-body exercise” with a focus on flexibility [14]. The normative values for Hong Kong for both, the sit-and-reach test and the back scratch test, are higher than ours [14]. This is also the case for the normative values of the sit-and-reach test for an older Taiwanese population [10]. Older men from Poland reached higher values in both flexibility tests while the values from older Polish women are similar to ours [16]. Apart from older men in one Spanish study [12], normative values from Spain and Portugal are lower for both flexibility tests [11–13]. Chilean women reached similar values in the sit-and-reach test and lower values in the back scratch test [15]. For the USA, Rikli & Jones reported a narrower range of values with higher values in the lower percentiles and lower values in the higher percentiles for the sit-and-reach test and overall higher values for the back scratch test [9].

The representativeness of the study sample for the older German population must be considered with caution. In comparison to national census data of 60–69-year-olds in 2011 [35] the study sample was better educated (22.0% (census 2011) vs. 31.6% (OUTDOOR ACTIVE) with Abitur (German equivalent to a high school diploma). Since there is a positive association between education and physical fitness [36], this could lead to an overestimation of normative values. All participants lived in Bremen, a city in the northwest of Germany. According to previously published studies, health-related behaviour e.g. physical activity differs between urban and rural areas [37, 38], however, there is yet no clear picture in which direction. Moreover, the included sub-districts in Bremen are highly heterogeneous. This is also reflected in the land use mix. Proportions of agricultural land use of the included sub-districts range from 0% (Neustadt and Ostertor) to 59.8% (Arbergen), thus a range of diverse areas is covered in the sample. Although disparities are slowly diminishing in the third decade after German reunification, prevalence of overweight and obesity still differs systematically between East and West Germany [39], and also body height is geographically patterned in Germany [40]. This might lead to overestimation of handgrip normative values and underestimation of chair stand normative values for Germany. In our study, we excluded institutionalized persons. In the age group 65 to 75 years, only a small proportion (1.11%) is in residential care [41], therefore, the normative values are probably not seriously impacted by this limitation. The OUTDOOR ACTIVE participants were able to participate in the study without taking part in the physical fitness tests. Participants of the physical fitness test were less likely to report only medium or poor subjective health compared to the other 433 survey participants (14.9% versus 26.3%) and less likely to be under constant medication (72.9% versus 76.7%) leading to a probable overestimation of normative values. This is a well-known limitation also in other studies [42]. One particular strength of the study is the use of the GAMLSS method, which gives normative values for each year of life and not only age groups.

## Conclusions

The present study is, to the best of our knowledge, the first to provide normative values for handgrip strength and components of the Senior Fitness Test in older adults aged 65 to 75 years residing in Germany. They might be useful in future research by providing evidence-based meaningful cut-offs for the investigated measures. Furthermore, they can be used for categorizing test results in (non-)clinical practice, and thus, supporting elaborated feedback to test participants.

## Supplementary Information

**Additional file 1.** Response variable distributions of the included models (selection based on the Akaike information criterion).

**Additional file 2.** Number of participants by sex and age.

**Additional file 3.** Means, standard deviations, and standard errors for all physical fitness measurements by sex and age.

**Additional file 4.** Tabulated sex- and age-specific normative values for handgrip strength (kg) (A), the 30s-chair stand test (n in 30s) (B), the 2 min-step test (n in 2 min) (C), the sit-and-reach test (cm) (D), and the back scratch test (cm) (E).

## Data Availability

The datasets used and/or analysed during the current study are available from the corresponding author on reasonable request.

## Abbreviations

GAMLSS: Generalized additive models for location, scale, and shape
ISCED: International Standard Classification of Education
