# Supplementary material for: Sex- and age-specific normative values for handgrip strength and components of the Senior Fitness Test in community-dwelling older adults aged 65–75 years in Germany: results from the OUTDOOR ACTIVE study
Source: BMC Geriatr. 2021 Apr 26;21:273. doi: 10.1186/s12877-021-02188-9 (PMC8074447; doi:10.1186/s12877-021-02188-9)
Supplement: Supplementary file 1 — Additional file 1. Response variable distributions of the included models (selection based on the Akaike information criterion). [file 12877_2021_2188_MOESM1_ESM.docx]

**Additional file 1** Response variable distributions of the included models (selection based on the Akaike information criterion).

| Physical fitness measurement | Women | Men |
| --- | --- | --- |
| Handgrip strength | TF2  (t family type 2) | GT  (generalised t) |
| 30s-chair stand test | exGAUS  (exponential Gaussian) | SHASHo  (sinh-arcsinh original) |
| 2 min-step test | BCT  (Box-Cox t) | LO  (logistic) |
| Sit-and-reach test | SEP2  (skew power exponential t2) | SN2  (skew normal type 2) |
| Back scratch test | SN2  (skew normal type 2) | SEP3  (skew power exponential t3) |
