# Supplementary material for: Sex- and age-specific normative values for handgrip strength and components of the Senior Fitness Test in community-dwelling older adults aged 65–75 years in Germany: results from the OUTDOOR ACTIVE study
Source: BMC Geriatr. 2021 Apr 26;21:273. doi: 10.1186/s12877-021-02188-9 (PMC8074447; doi:10.1186/s12877-021-02188-9)
Supplement: Supplementary file 2 — Additional file 2. Number of participants by sex and age. [file 12877_2021_2188_MOESM2_ESM.docx]

**Additional file 2** Number of participants by sex and age.

| Age  (years) | Women (n=880) | Men (n=777) |
| --- | --- | --- |
|  | n (%) | n (%) |
| 65 | 47 (5.3) | 37 (4.8) |
| 66 | 122 (13.9) | 98 (12.6) |
| 67 | 96 (10.9) | 98 (12.6) |
| 68 | 96 (10.9) | 99 (12.7) |
| 69 | 97 (11.0) | 89 (11.5) |
| 70 | 78 (8.9) | 73 (9.4) |
| 71 | 94 (10.7) | 69 (8.9) |
| 72 | 83 (9.4) | 76 (9.8) |
| 73 | 62 (7.0) | 46 (5.9) |
| 74 | 65 (7.4) | 63 (8.1) |
| 75 | 40 (4.5) | 29 (3.7) |
|  |  |  |
