# Supplementary material for: Sex- and age-specific normative values for handgrip strength and components of the Senior Fitness Test in community-dwelling older adults aged 65–75 years in Germany: results from the OUTDOOR ACTIVE study
Source: BMC Geriatr. 2021 Apr 26;21:273. doi: 10.1186/s12877-021-02188-9 (PMC8074447; doi:10.1186/s12877-021-02188-9)
Supplement: Supplementary file 3 — Additional file 3. Means, standard deviations, and standard errors for all physical fitness measurements by sex and age. [file 12877_2021_2188_MOESM3_ESM.docx]

**Additional file 3** Means, standard deviations, and standard errors for all physical fitness measurements by sex and age.

| Women | | | | | | | | | | | | | | | |
| --- | --- | --- | --- | --- | --- | --- | --- | --- | --- | --- | --- | --- | --- | --- | --- |
| Age  (years) | Handgrip strength (kg) | | | 30s-chair stand test (n in 30s) | | | 2 min-step test (n in 2 min) | | | Sit-and-reach test (cm) | | | Back scratch test (cm) | | |
|  | Mean | SD | SE | Mean | SD | SE | Mean | SD | SE | Mean | SD | SE | Mean | SD | SE |
| 65 | 27.7 | 5.7 | 0.8 | 13.3 | 3.2 | 0.5 | 88.0 | 21.2 | 3.2 | 7.1 | 10.0 | 1.5 | -4.3 | 8.9 | 1.3 |
| 66 | 26.0 | 4.9 | 0.5 | 13.1 | 2.6 | 0.2 | 86.0 | 18.0 | 1.6 | 2.9 | 8.7 | 0.8 | -3.6 | 8.7 | 0.8 |
| 67 | 25.5 | 5.3 | 0.5 | 13.2 | 3.2 | 0.3 | 85.1 | 19.2 | 2.0 | 4.4 | 10.3 | 1.1 | -2.9 | 9.3 | 1.0 |
| 68 | 26.7 | 5.1 | 0.5 | 13.2 | 2.7 | 0.3 | 87.7 | 20.2 | 2.1 | 3.9 | 10.3 | 1.1 | -4.7 | 7.9 | 0.8 |
| 69 | 25.1 | 4.8 | 0.5 | 12.9 | 3.1 | 0.3 | 85.6 | 18.7 | 1.9 | 4.9 | 9.3 | 1.0 | -3.9 | 8.6 | 0.9 |
| 70 | 25.6 | 5.1 | 0.6 | 13.2 | 3.5 | 0.4 | 87.8 | 19.5 | 2.2 | 3.0 | 10.2 | 1.2 | -3.7 | 8.8 | 1.0 |
| 71 | 23.7 | 5.0 | 0.5 | 12.8 | 2.9 | 0.3 | 82.4 | 19.1 | 2.0 | 4.2 | 8.8 | 0.9 | -5.3 | 10.1 | 1.1 |
| 72 | 25.1 | 4.5 | 0.5 | 12.6 | 2.7 | 0.3 | 83.0 | 17.8 | 2.0 | 2.5 | 9.6 | 1.1 | -4.2 | 8.9 | 1.0 |
| 73 | 23.6 | 4.7 | 0.6 | 12.1 | 2.6 | 0.3 | 83.1 | 17.1 | 2.3 | 1.0 | 10.1 | 1.3 | -6.1 | 9.4 | 1.2 |
| 74 | 24.4 | 5.6 | 0.7 | 12.5 | 3.3 | 0.4 | 80.2 | 19.0 | 2.5 | 2.9 | 10.5 | 1.4 | -7.8 | 10.3 | 1.3 |
| 75 | 23.2 | 4.8 | 0.8 | 12.3 | 2.8 | 0.5 | 79.0 | 20.4 | 3.3 | 3.5 | 12.1 | 2.0 | -5.2 | 8.2 | 1.3 |
|  |  |  |  |  |  |  |  |  |  |  |  |  |  |  |  |
| Total | 25.2 | 5.1 | 0.2 | 12.9 | 3.0 | 0.1 | 84.7 | 17.7 | 0.7 | 3.6 | 9.9 | 0.3 | -4.5 | 9.0 | 0.3 |
|  |  |  |  |  |  |  |  |  |  |  |  |  |  |  |  |
| Men | | | | | | | | | | | | | | | |
| Age (years) | Handgrip strength (kg) | | | 30s-chair stand test (n in 30s) | | | 2 min-step test (n in 2 min) | | | Sit-and-reach test (cm) | | | Back scratch test (cm) | | |
|  | Mean | SD | SE | Mean | SD | SE | Mean | SD | SE | Mean | SD | SE | Mean | SD | SE |
| 65 | 43.7 | 8.1 | 1.3 | 13.8 | 3.0 | 0.5 | 91.7 | 15.1 | 2.5 | -3.7 | 9.7 | 1.6 | -10.4 | 10.5 | 1.6 |
| 66 | 43.9 | 8.6 | 0.9 | 13.7 | 3.3 | 0.3 | 88.4 | 18.7 | 1.9 | -3.5 | 12.5 | 1.3 | -11.2 | 13.4 | 1.3 |
| 67 | 43.7 | 8.4 | 0.9 | 13.4 | 2.5 | 0.3 | 88.3 | 16.1 | 1.6 | -2.6 | 11.9 | 1.2 | -9.0 | 13.3 | 1.2 |
| 68 | 43.6 | 7.0 | 0.7 | 13.7 | 3.0 | 0.3 | 88.1 | 15.4 | 1.6 | -3.9 | 10.7 | 1.1 | -12.2 | 12.1 | 1.1 |
| 69 | 41.2 | 8.0 | 0.8 | 13.4 | 3.5 | 0.4 | 85.7 | 21.9 | 2.3 | -3.3 | 10.6 | 1.1 | -8.9 | 10.3 | 1.1 |
| 70 | 43.1 | 7.0 | 0.8 | 13.7 | 3.0 | 0.4 | 90.1 | 16.2 | 1.9 | -3.4 | 10.1 | 1.2 | -12.5 | 12.2 | 1.2 |
| 71 | 40.8 | 7.1 | 0.9 | 13.1 | 2.7 | 0.3 | 88.9 | 18.4 | 2.3 | -5.0 | 10.8 | 1.3 | -11.6 | 11.3 | 1.3 |
| 72 | 39.7 | 7.7 | 0.9 | 13.2 | 3.2 | 0.4 | 85.2 | 15.9 | 1.8 | -3.8 | 12.3 | 1.4 | -12.5 | 12.9 | 1.4 |
| 73 | 40.4 | 7.1 | 1.1 | 13.1 | 3.4 | 0.5 | 83.2 | 19.1 | 2.8 | -6.2 | 12.8 | 1.9 | -13.8 | 13.2 | 1.9 |
| 74 | 39.4 | 7.0 | 0.9 | 13.0 | 2.7 | 0.3 | 83.9 | 15.5 | 2.0 | -5.8 | 11.1 | 1.4 | -13.3 | 13.1 | 1.4 |
| 75 | 39.8 | 8.2 | 1.5 | 13.9 | 3.5 | 0.7 | 80.5 | 22.1 | 4.1 | -2.3 | 11.7 | 2.2 | -16.0 | 13.3 | 2.2 |
|  |  |  |  |  |  |  |  |  |  |  |  |  |  |  |  |
| Total | 42.0 | 7.8 | 0.3 | 13.4 | 3.0 | 0.1 | 87.1 | 17.7 | 0.6 | -3.9 | 11.3 | 0.4 | -11.6 | 12.4 | 0.5 |
|  |  |  |  |  |  |  |  |  |  |  |  |  |  |  |  |

SD standard deviation

SE standard error
