# Supplementary material for: Sex- and age-specific normative values for handgrip strength and components of the Senior Fitness Test in community-dwelling older adults aged 65–75 years in Germany: results from the OUTDOOR ACTIVE study
Source: BMC Geriatr. 2021 Apr 26;21:273. doi: 10.1186/s12877-021-02188-9 (PMC8074447; doi:10.1186/s12877-021-02188-9)
Supplement: Supplementary file 4 — Additional file 4. Tabulated sex- and age-specific normative values for handgrip strength (kg) (A), the 30s-chair stand test (n in 30s) (B), the 2 min-step test (n in 2 min) (C), the sit-and-reach test (cm) (D), and the back scratch test (cm) (E). [file 12877_2021_2188_MOESM4_ESM.docx]

**Additional file 4** Tabulated sex- and age-specific normative values for handgrip strength (kg) (A), the 30s-chair stand test (n in 30s) (B), the 2 min-step test (n in 2 min) (C), the sit-and-reach test (cm) (D), and the back scratch test (cm) (E).

**A** Sex- and age-specific normative values for handgrip strength (kg).

|  | Percentiles for women (n=880) | | | | | | | | |
| --- | --- | --- | --- | --- | --- | --- | --- | --- | --- |
| Age (years) | 1 | 3 | 10 | 25 | 50 | 75 | 90 | 97 | 99 |
| 65 | 13.7 | 16.6 | 20.1 | 23.5 | 27.0 | 30.5 | 33.8 | 37.3 | 40.3 |
| 66 | 13.5 | 16.3 | 19.7 | 22.9 | 26.3 | 29.7 | 32.8 | 36.3 | 39.1 |
| 67 | 13.3 | 16.1 | 19.5 | 22.6 | 26.0 | 29.3 | 32.4 | 35.8 | 38.6 |
| 68 | 13.4 | 16.2 | 19.5 | 22.6 | 25.8 | 29.1 | 32.2 | 35.5 | 38.3 |
| 69 | 13.2 | 15.9 | 19.2 | 22.2 | 25.5 | 28.7 | 31.7 | 35.0 | 37.7 |
| 70 | 12.9 | 15.6 | 18.8 | 21.8 | 25.0 | 28.2 | 31.2 | 34.4 | 37.1 |
| 71 | 12.8 | 15.4 | 18.5 | 21.4 | 24.6 | 27.7 | 30.6 | 33.8 | 36.4 |
| 72 | 12.8 | 15.4 | 18.5 | 21.3 | 24.4 | 27.4 | 30.3 | 33.4 | 35.9 |
| 73 | 12.5 | 15.1 | 18.1 | 21.0 | 24.1 | 27.1 | 30.0 | 33.1 | 35.7 |
| 74 | 12.0 | 14.6 | 17.7 | 20.7 | 23.8 | 26.9 | 29.9 | 33.0 | 35.6 |
| 75 | 11.4 | 14.1 | 17.3 | 20.3 | 23.4 | 26.6 | 29.6 | 32.8 | 35.4 |
|  |  |  |  |  |  |  |  |  |  |
|  | Percentiles for men (n=777) | | | | | | | | |
| Age (years) | 1 | 3 | 10 | 25 | 50 | 75 | 90 | 97 | 99 |
| 65 | 24.9 | 28.8 | 33.7 | 38.4 | 44.0 | 49.7 | 54.4 | 59.3 | 63.2 |
| 66 | 25.1 | 29.0 | 33.8 | 38.5 | 44.0 | 49.6 | 54.3 | 59.1 | 63.0 |
| 67 | 25.3 | 29.1 | 33.7 | 37.3 | 43.7 | 49.1 | 53.7 | 58.3 | 62.1 |
| 68 | 25.3 | 29.0 | 33.4 | 37.9 | 43.1 | 48.3 | 52.7 | 57.2 | 60.9 |
| 69 | 24.7 | 28.3 | 32.8 | 37.1 | 42.3 | 47.5 | 51.8 | 56.3 | 59.9 |
| 70 | 24.6 | 28.2 | 32.5 | 36.7 | 41.8 | 46.8 | 51.0 | 55.4 | 58.9 |
| 71 | 24.0 | 27.5 | 31.8 | 36.0 | 40.9 | 45.9 | 50.1 | 54.3 | 57.8 |
| 72 | 23.2 | 26.7 | 31.0 | 35.2 | 40.2 | 45.2 | 49.5 | 53.8 | 57.3 |
| 73 | 22.8 | 26.3 | 30.6 | 34.8 | 39.8 | 44.8 | 49.1 | 53.4 | 56.9 |
| 74 | 22.4 | 25.9 | 30.3 | 34.5 | 39.6 | 44.6 | 48.9 | 53.2 | 56.8 |
| 75 | 21.6 | 25.2 | 29.7 | 34.1 | 39.3 | 44.6 | 49.0 | 53.4 | 57.1 |
|  |  |  |  |  |  |  |  |  |  |

**B** Sex- and age-specific normative values for the 30s-chair stand test (n in 30s).

|  | Percentiles for women (n=854) | | | | | | | | |
| --- | --- | --- | --- | --- | --- | --- | --- | --- | --- |
| Age (years) | 1 | 3 | 10 | 25 | 50 | 75 | 90 | 97 | 99 |
| 65 | 6.5 | 7.8 | 9.5 | 11.3 | 13.2 | 15.2 | 16.9 | 18.7 | 20.0 |
| 66 | 6.6 | 7.8 | 9.5 | 11.2 | 13.2 | 15.1 | 16.8 | 18.5 | 19.8 |
| 67 | 6.4 | 7.7 | 9.4 | 11.2 | 13.1 | 15.1 | 16.9 | 18.6 | 19.9 |
| 68 | 6.2 | 7.5 | 9.3 | 11.1 | 13.1 | 15.1 | 16.9 | 18.7 | 20.0 |
| 69 | 5.8 | 7.1 | 9.0 | 10.8 | 12.9 | 15.0 | 16.8 | 18.7 | 20.0 |
| 70 | 5.6 | 7.0 | 8.9 | 10.8 | 12.8 | 14.9 | 16.8 | 18.7 | 20.0 |
| 71 | 5.9 | 7.2 | 9.0 | 10.8 | 12.8 | 14.8 | 16.6 | 18.4 | 19.7 |
| 72 | 6.0 | 7.2 | 9.0 | 10.7 | 12.6 | 14.5 | 16.2 | 17.9 | 19.2 |
| 73 | 5.8 | 7.1 | 8.8 | 10.5 | 12.4 | 14.3 | 16.0 | 17.7 | 19.0 |
| 74 | 5.6 | 6.9 | 8.6 | 10.4 | 12.3 | 14.3 | 16.1 | 17.8 | 19.1 |
| 75 | 5.4 | 6.7 | 8.5 | 10.3 | 12.3 | 14.3 | 16.1 | 17.9 | 19.2 |
|  |  |  |  |  |  |  |  |  |  |
|  | Percentiles for men (n=758) | | | | | | | | |
| Age (years) | 1 | 3 | 10 | 25 | 50 | 75 | 90 | 97 | 99 |
| 65 | 6.7 | 8.2 | 10.0 | 11.8 | 13.6 | 15.6 | 17.8 | 20.2 | 22.2 |
| 66 | 6.8 | 8.2 | 10.0 | 11.7 | 13.4 | 15.4 | 17.5 | 19.8 | 21.7 |
| 67 | 7.0 | 8.4 | 10.1 | 11.7 | 13.3 | 15.2 | 17.2 | 19.5 | 21.3 |
| 68 | 6.7 | 8.2 | 10.0 | 11.6 | 13.3 | 15.3 | 17.3 | 19.7 | 21.6 |
| 69 | 6.4 | 7.9 | 9.8 | 11.5 | 13.3 | 15.3 | 17.5 | 20.0 | 22.0 |
| 70 | 6.4 | 7.9 | 9.8 | 11.5 | 13.2 | 15.2 | 17.4 | 19.8 | 21.8 |
| 71 | 6.5 | 7.9 | 9.7 | 11.3 | 13.1 | 15.0 | 17.1 | 19.4 | 21.3 |
| 72 | 6.3 | 7.7 | 9.5 | 11.2 | 13.0 | 14.9 | 17.0 | 19.4 | 21.3 |
| 73 | 6.3 | 7.7 | 9.5 | 11.2 | 13.0 | 14.9 | 17.0 | 19.4 | 21.3 |
| 74 | 6.4 | 7.9 | 9.7 | 11.3 | 13.0 | 14.9 | 17.0 | 19.4 | 21.3 |
| 75 | 6.4 | 7.9 | 9.7 | 11.4 | 13.2 | 15.1 | 17.3 | 19.7 | 21.7 |
|  |  |  |  |  |  |  |  |  |  |

**C** Sex- and age-specific normative values for the 2 min-step test (n in 2 min).

|  | Percentiles for women (n=852) | | | | | | | | |
| --- | --- | --- | --- | --- | --- | --- | --- | --- | --- |
| Age (years) | 1 | 3 | 10 | 25 | 50 | 75 | 90 | 97 | 99 |
| 65 | 33.3 | 46.7 | 62.3 | 75.6 | 88.7 | 100.8 | 111.4 | 122.2 | 130.5 |
| 66 | 34.9 | 47.8 | 62.5 | 75.1 | 87.5 | 99.0 | 109.1 | 119.4 | 127.4 |
| 67 | 34.7 | 47.6 | 62.3 | 74.9 | 87.3 | 98.8 | 108.9 | 119.1 | 127.1 |
| 68 | 34.2 | 47.2 | 62.2 | 75.0 | 87.6 | 99.3 | 109.6 | 119.6 | 128.0 |
| 69 | 33.6 | 46.8 | 61.8 | 74.7 | 87.4 | 99.1 | 109.4 | 119.9 | 128.0 |
| 70 | 33.1 | 46.1 | 61.1 | 74.0 | 86.6 | 98.3 | 108.6 | 119.0 | 127.1 |
| 71 | 32.8 | 45.6 | 60.3 | 72.8 | 85.2 | 96.6 | 106.6 | 116.8 | 124.7 |
| 72 | 32.9 | 45.4 | 59.7 | 71.9 | 83.9 | 95.0 | 104.8 | 114.7 | 122.4 |
| 73 | 31.8 | 44.2 | 58.5 | 70.7 | 82.7 | 93.8 | 103.6 | 113.5 | 121.2 |
| 74 | 28.5 | 41.1 | 56.1 | 68.9 | 81.4 | 93.0 | 103.1 | 113.4 | 121.3 |
| 75 | 24.4 | 37.2 | 53.0 | 66.7 | 80.1 | 92.3 | 103.1 | 113.9 | 122.3 |
|  |  |  |  |  |  |  |  |  |  |
|  | Percentiles for men (n=764) | | | | | | | | |
| Age (years) | 1 | 3 | 10 | 25 | 50 | 75 | 90 | 97 | 99 |
| 65 | 48.7 | 59.0 | 70.8 | 80.9 | 91.0 | 101.1 | 111.2 | 122.9 | 133.2 |
| 66 | 45.6 | 56.3 | 68.6 | 79.1 | 89.7 | 100.2 | 110.8 | 123.0 | 133.8 |
| 67 | 44.7 | 55.4 | 67.7 | 78.2 | 88.7 | 99.2 | 109.7 | 122.0 | 132.7 |
| 68 | 43.4 | 54.3 | 66.7 | 77.3 | 88.0 | 98.7 | 109.4 | 121.8 | 132.6 |
| 69 | 41.4 | 52.7 | 65.6 | 76.7 | 87.8 | 98.9 | 110.0 | 122.9 | 134.2 |
| 70 | 42.3 | 53.5 | 66.2 | 77.2 | 88.1 | 99.1 | 110.0 | 122.8 | 134.0 |
| 71 | 42.9 | 53.8 | 66.2 | 76.9 | 87.6 | 98.3 | 108.9 | 121.4 | 132.3 |
| 72 | 42.2 | 52.9 | 65.1 | 75.5 | 86.0 | 96.4 | 106.9 | 119.0 | 129.7 |
| 73 | 40.5 | 51.2 | 63.4 | 73.9 | 84.4 | 94.9 | 105.4 | 117.6 | 128.3 |
| 74 | 37.8 | 48.8 | 61.4 | 72.2 | 83.0 | 93.8 | 104.6 | 117.2 | 128.2 |
| 75 | 32.4 | 44.4 | 58.0 | 69.7 | 81.4 | 93.1 | 104.8 | 118.4 | 130.4 |
|  |  |  |  |  |  |  |  |  |  |

**D** Sex- and age-specific normative values for the sit-and-reach test (cm).

|  | Percentiles for women (n=851) | | | | | | | | |
| --- | --- | --- | --- | --- | --- | --- | --- | --- | --- |
| Age (years) | 1 | 3 | 10 | 25 | 50 | 75 | 90 | 97 | 99 |
| 65 | -18.4 | -12.9 | -6.3 | -0.4 | 5.0 | 10.8 | 17.0 | 24.0 | 29.7 |
| 66 | -19.7 | -14.2 | -7.6 | -1.8 | 3.6 | 9.4 | 15.6 | 22.5 | 28.2 |
| 67 | -20.8 | -15.1 | -8.3 | -2.2 | 3.4 | 9.4 | 15.8 | 23.1 | 28.9 |
| 68 | -20.9 | -15.1 | -8.2 | -2.0 | 3.7 | 9.7 | 16.2 | 23.5 | 29.5 |
| 69 | -20.3 | -14.6 | -7.8 | -1.8 | 3.8 | 9.8 | 16.2 | 23.3 | 29.2 |
| 70 | -19.8 | -14.4 | -7.7 | -1.8 | 3.7 | 9.5 | 15.7 | 22.7 | 28.4 |
| 71 | -19.7 | -14.3 | -7.8 | -2.0 | 3.4 | 9.1 | 15.2 | 22.1 | 27.7 |
| 72 | -21.0 | -15.4 | -8.7 | -2.7 | 2.8 | 8.7 | 14.9 | 22.0 | 27.8 |
| 73 | -22.6 | -16.8 | -9.7 | -3.4 | 2.4 | 8.6 | 15.2 | 22.6 | 28.7 |
| 74 | -24.0 | -17.8 | -10.3 | -3.7 | 2.5 | 9.1 | 16.1 | 24.0 | 30.4 |
| 75 | -25.5 | -18.8 | -10.8 | -3.7 | 2.9 | 10.0 | 17.5 | 26.0 | 32.9 |
|  |  |  |  |  |  |  |  |  |  |
|  | Percentiles for men (n=753) | | | | | | | | |
| Age (years) | 1 | 3 | 10 | 25 | 50 | 75 | 90 | 97 | 99 |
| 65 | -30.6 | -25.2 | -18.0 | -10.8 | -3.1 | 3.9 | 9.9 | 15.6 | 19.8 |
| 66 | -32.2 | -26.4 | -18.7 | -11.0 | -2.8 | 4.7 | 11.1 | 17.2 | 21.7 |
| 67 | -31.9 | -26.2 | -18.5 | -10.8 | -2.7 | 4.8 | 11.1 | 17.2 | 21.7 |
| 68 | -30.5 | -25.1 | -17.8 | -10.6 | -2.9 | 4.2 | 10.1 | 15.9 | 20.1 |
| 69 | -29.6 | -24.3 | -17.4 | -10.4 | -3.0 | 3.8 | 9.5 | 15.0 | 19.1 |
| 70 | -29.9 | -24.6 | -17.7 | -10.7 | -3.3 | 3.5 | 9.2 | 14.8 | 18.9 |
| 71 | -31.7 | -26.2 | -18.8 | -11.6 | -3.8 | 3.3 | 9.4 | 15.2 | 19.4 |
| 72 | -33.9 | -28.0 | -20.2 | -12.5 | -4.2 | 3.3 | 9.7 | 15.9 | 20.5 |
| 73 | -34.9 | -28.9 | -21.0 | -13.1 | -4.6 | 3.1 | 9.7 | 16.0 | 20.6 |
| 74 | -34.2 | -28.3 | -20.5 | -12.7 | -4.4 | 3.3 | 9.7 | 15.9 | 20.5 |
| 75 | -32.8 | -27.0 | -19.3 | -11.6 | -3.4 | 4.2 | 10.5 | 16.7 | 21.2 |
|  |  |  |  |  |  |  |  |  |  |

**E** Sex- and age-specific normative values for the back scratch test (cm).

|  | Percentiles for women (n=851) | | | | | | | | |
| --- | --- | --- | --- | --- | --- | --- | --- | --- | --- |
| Age (years) | 1 | 3 | 10 | 25 | 50 | 75 | 90 | 97 | 99 |
| 65 | -29.0 | -23.6 | -16.4 | -9.6 | -2.7 | 2.9 | 6.4 | 9.4 | 11.4 |
| 66 | -27.8 | -22.6 | -15.8 | -9.2 | -2.7 | 2.6 | 6.0 | 8.8 | 10.8 |
| 67 | -27.3 | -22.3 | -15.6 | -9.1 | -2.7 | 2.5 | 5.8 | 8.6 | 10.6 |
| 68 | -27.5 | -22.4 | -15.7 | -9.3 | -2.9 | 2.3 | 5.6 | 8.4 | 10.3 |
| 69 | -28.2 | -23.0 | 16.1 | -9.5 | -2.9 | 2.5 | 5.9 | 8.7 | 10.7 |
| 70 | -29.3 | -23.8 | -16.7 | -9.9 | -3.0 | 2.5 | 6.1 | 9.0 | 11.1 |
| 71 | -30.4 | -24.8 | -17.5 | -10.4 | -3.3 | 2.3 | 6.0 | 9.0 | 11.2 |
| 72 | -31.1 | -25.5 | -18.1 | -10.9 | -3.7 | 2.0 | 5.7 | 8.8 | 10.9 |
| 73 | -32.2 | -26.5 | -19.0 | -11.7 | -4.4 | 1.4 | 5.1 | 8.2 | 10.4 |
| 74 | -32.5 | -26.8 | -19.4 | -12.2 | -4.9 | 0.8 | 4.5 | 7.6 | 9.8 |
| 75 | -31.0 | -25.6 | -18.5 | -11.7 | -4.9 | 0.6 | 4.1 | 7.1 | 9.1 |
|  |  |  |  |  |  |  |  |  |  |
|  | Percentiles for men (n=753) | | | | | | | | |
| Age (years) | 1 | 3 | 10 | 25 | 50 | 75 | 90 | 97 | 99 |
| 65 | -37.4 | -32.9 | -26.2 | -18.8 | -9.8 | -1.4 | 4.3 | 8.7 | 11.5 |
| 66 | -40.3 | -35.3 | -28.1 | -20.1 | -10.4 | -1.3 | 4.8 | 9.6 | 12.6 |
| 67 | -40.7 | -35.7 | -28.5 | -20.3 | -10.6 | -1.4 | 4.8 | 9.6 | 12.6 |
| 68 | -39.1 | -34.4 | -27.5 | -19.8 | -10.4 | -1.7 | 4.2 | 8.8 | 11.7 |
| 69 | -37.7 | -33.2 | -26.5 | -19.1 | -10.1 | -1.7 | 4.0 | 8.4 | 11.2 |
| 70 | -38.4 | -33.8 | -27.1 | -19.6 | -10.5 | -2.0 | 3.7 | 8.1 | 11.0 |
| 71 | -39.7 | -35.0 | -28.1 | -20.5 | -11.2 | -2.5 | 3.3 | 7.8 | 10.7 |
| 72 | -41.5 | -36.6 | -29.5 | -21.6 | -12.0 | -3.0 | 3.0 | 7.7 | 10.7 |
| 73 | -43.1 | -38.1 | -30.8 | -22.6 | -12.7 | -3.5 | 2.8 | 7.6 | 10.7 |
| 74 | -44.6 | -39.4 | -31.9 | -23.5 | -13.3 | -3.8 | 2.6 | 7.6 | 10.7 |
| 75 | -46.4 | -41.0 | -33.3 | -24.6 | -14.1 | -4.3 | 2.3 | 7.5 | 10.8 |
|  |  |  |  |  |  |  |  |  |  |
